# Supplementary material for: Post-synthetic modification of covalent organic frameworks for CO2 electroreduction
Source: Nat Commun. 2023 Jun 26;14:3800. doi: 10.1038/s41467-023-39544-9 (PMC10293238; doi:10.1038/s41467-023-39544-9)
Supplement: Supplementary file 1 — Supplementary Information [file 41467_2023_39544_MOESM1_ESM.pdf]

Supplementary Information

Post-synthetic modification of covalent organic frameworks for CO_2 electroreduction

Minghao Liu[1,2]†, Shuai Yang[1,3]†, Xiubei Yang[1,4], Cheng-Xing Cui[5,6],* Guojuan Liu[1,4], Xuewen Li[1,4], Jun He[2], George Zheng Chen[7], Qing Xu[1,4]* & Gaofeng Zeng[1,4]*

1 CAS Key Laboratory of Low-Carbon Conversion Science and Engineering, Shanghai Advanced Research Institute, Chinese Academy of Sciences, Shanghai, 201210, P. R. China

2 Department of Chemical and Environmental Engineering, University of Nottingham Ningbo China, Ningbo 315199, P.R. China

3 School of Physical Science and Technology, ShanghaiTech University, Shanghai 201210, P. R. China

4 School of Chemical Engineering, University of Chinese Academy of Sciences, Beijing 100049, P. R. China

5 School of Chemistry and Chemical Engineering, Institute of Computational Chemistry, Henan Institute of Science and Technology, Xinxiang 453003, P. R. China

6 ZhengZhou JiShu Institute of AI Science, Zhengzhou 451162, P. R. China

7 Department of Chemical and Environmental Engineering, University of Nottingham, Nottingham NG7 2RD, UK

† These authors contributed equally to this work

* Corresponding authors:

C. C.: chengxingcui@hist.edu.cn; Q. X.: xuqing@sari.ac.cn; G. Z.: zenggf@sari.ac.cn

Supplementary Methods

Materials.

All the chemicals commercially available were used without further purification. 5,10,15,20-tetrakis(4-aminophenyl)porphinato]-cobalt (TAPP(Co), >97%), 4,4',4'',4'''-(1,4-phenylenebis(azanetriyl))tetrabenzaldehyde (PATA, >97%), iodomethane (CH_3I, >98%) and $NaBH_4$ (>99%) were obtained from Alfa. Acetic acid, o-Dichlorobenzene (o-DCB, >99%), tetrahydrofuran (THF, >97%), n-Butanol (BuOH, >97%) and Dimethylacetamide (DMAC, >98%) were obtained from Sinopharm Chemical Reagent Co.,Ltd.

Pawley refinement.

The powder X-ray diffraction (PXRD) pattern simulation was performed using a software package for crystal determination from PXRD pattern, implemented in Reflex module of Materials Studio. We performed Pawley refinement to optimize the lattice parameters iteratively until the RP and RWP values converge. The pseudo-Voigt profile function was used for whole profile fitting and Finger-Cox-Jephcoat function was used for asymmetry correction during the refinement processes.

Electrochemical performance tests.

The preparation of working electrode 5 mg COFs and 8 mg carbon black was grinded for 10 min and dispersed in mixed solution of 50 μL Nafion solution (5 wt%), ethanol (950 μL) followed with sonication for 120 min. The 320 μL as-prepared catalyst ink was directly spray-coated on a hydrophobic carbon paper (2 cm × 2 cm) to form a 4 cm^2 catalyst area with a catalyst loading of 0.4 mg cm^{-2}, and the active material was 3.6, 3.5, 3.3, and 3.1 wt.% for CoTAPP-PATA-COF, N^+-COF, NH-COF and N^+-NH-COF. In addition, the carbon paper with the thickness was 0.37 mm. The deposited carbon paper was further dried at room temperature. All the electrochemical experiments were performed in a H-type cell with two-compartments separated by an anion exchange membrane (Nafion-117). Each compartment contained 70 mL electrolyte (0.5 M $KHCO_3$). Electrochemical measurements were performed in a three-electrode cell using the

Ag/AgCl electrode as the reference electrode and Pt foil as the counter electrode on the electrochemical instrument (CHI760E). Before the electrochemical measurements, the electrolyte solution was purged with CO_2 for 30 min to obtain the CO_2-saturated solution. The pH is 7.2 for CO_2-saturated 0.5 M $KHCO_3$ and 8.8 N_2-saturated 0.5 M $KHCO_3$. A mass flow controller was used to set the N_2 or CO_2 flow rate at 30. The LSV curves were conducted with scan rate of 10 mV/s. All the potentials were reported with respect to the reversible hydrogen electrode (RHE) and conversed using the formula E (vs. RHE) = E (vs. Ag/AgCl) + 0.196 V +0.059 × pH. Gas products were detected by gas chromatograph.

The calculation of Faradic efficiency.

$$FE = \frac{N \times F \times n_{CO}}{I \times 60\ (s/min)} \times 100\% \quad (1)$$

Where F is the Faraday constant; n_{CO} is the moles of produced CO; I is total steady-state cell current; N is the electron transfer number for product formation.

Turnover frequency (TOF).

The turnover frequency (TOF) was evaluated by the following standard equation:

$$TOF = (J \times A)/(2 \times F \times n) \quad (2)$$

Where j (A/cm^2) is the partial current for certain product; A is the geometric surface area of the electrode; F stands for the Faraday constant; n (mol) is molar amount of cobalt loaded on the GC electrode which was determined by the ICP analysis.

All metal cations in the COFs were assumed to be catalytically active, so the calculated value represents the lower limits of the TOF.

Mott-Schottky curve test. Preparation of working electrode: 2 mg COF powder was mixed with 1.0 mL ethanol and 50 uL Nafion (5%) dispersion, and ultrasonic treatment was performed for 30 min. The resulting mixture slurry is evenly coated on the bottom of the ITO glass plate in the 1 × 2 cm^2 area, and placed in the air to stand for drying naturally.

Electrochemical test for Mott-Schottky curve.

Electrochemical measurements were performed in a three-electrode cell using the Ag/AgCl electrode as the reference electrode. Using 500 Hz and 1000 Hz to test the curve.

Characterizations.

Powder X-ray diffraction (PXRD) data were recorded on a Rigaku model RINT Ultima III diffractometer by depositing powder on glass substrate, from 2θ = 2° up to 60° with 0.02° increment. Nitrogen sorption isotherms were measured at 77 K with a Micromeritics Instrument Corporation model 3Flex surface characterization analyzer. The Brunauer-Emmett-Teller (BET) method was utilized to calculate the specific surface areas. By using the non-local density functional theory (NLDFT) model, the pore volume was derived from the sorption curve. FE-SEM images were obtained on a FEI Sirion-200 or Hitachi high technologies (SU-6600) field-emission scanning electron microscope at an electric voltage of 5 KV. EDX and elemental mapping were acquired using a HITCHI Miniscope TM3030. High-resolution transmission electron microscope images were obtained by transmission electron microscopy (TEM, FEI Tecnai G2). ICP was performed on a Perkin-Elmer Elan DRC II Quadrupole Inductively Coupled Plasma Mass Spectrometer (ICP-MS) analyzer. Fourier transform infrared (FT-IR) spectroscopy was measured using KBr pellets on a Perkin Elmer Spectrum 100 spectrometer in the 500-4000 cm^{-1}. UV-vis absorbance spectra were recorded on a Cary 5000 UV-vis-NIR spectrophotometer equipped with a mercury lamp. Substrates were mixed with $BaSO_4$. Base line correction was done towards $BaSO_4$. Spectra were taken over a wavelength range of 200-800 nm with a 5 min collection time and 1 nm resolution. The cobalt content of samples was obtained using inductively coupled plasma optical emission spectroscopy (ICP-OES) on an Optima 8000 spectrometer.

DFT calculation.

Density-functional theory simulation: The cluster model was adopted to perform the density-functional theory (DFT) calculation, where a piece of “cluster” structure was taken from the periodic boundary model of each four COFs. The cluster models include 134, 136, 142, 144

atoms for CoTAPP-PATA-COF, NH-COF, N^+-COF and N^+-NH-COF, respectively. The CAM-B3-LYP functional with the standard 6-311G(d) basis set and the SDD effective core potential for Co, which was denoted as CAM-B3LYP/6-311G(d) (SDD for Co), was used for the optimization of reactants, intermediates and products without any restriction. Frequency calculations were further carried out for all stationary points to determine whether they are local minima or transition states and to obtain the thermochemical corrections for enthalpies and Gibbs free energies. All calculations were performed with Gaussian 16. The software of Multiwfn was further used to obtain the total density of states (TDOSs), partitional density of states (PDOSs) of Co, COOH and remainder of COOH* for CoTAPP-PATA-COF, N^+-COF, NH-COF and N^+-NH-COF catalyzing CO_2RR.[1]

These are the equations of the free energy (G) for different steps in the calculation method:

(1) $*+CO_2 \rightarrow *CO_2$ $\qquad G_1=G_{*CO2}-G_{*}-G_{CO2}$ (3)

(2) $*+CO_2+H^++e^- \rightarrow *COOH$ $\qquad G_2=G_{*COOH}-G^*-G_{CO2}-G_{H+}$-eU (4)

(3) $*+CO_2+2H^++2e^- \rightarrow *CO+H_2O$ $\qquad G_3=G_{*CO}+G_{H2O}-G^*-G_{CO2}-2G_{H+}$-2eU (5)

(4) $*+CO_2+2H^++2e^- \rightarrow *+CO+H_2O$ $\qquad G_4=G_{*}+G_{CO}+G_{H2O}-G_{*}-G_{CO2}-2G_{H+}$-2eU (6)

And the free energy changes (ΔG) values of every step (ΔG_2, ΔG_3 and ΔG_4) were calculated from the value of G_2-G_1, G_3-G_2 and G_4-G_3, respectively.

Supplementary Figures

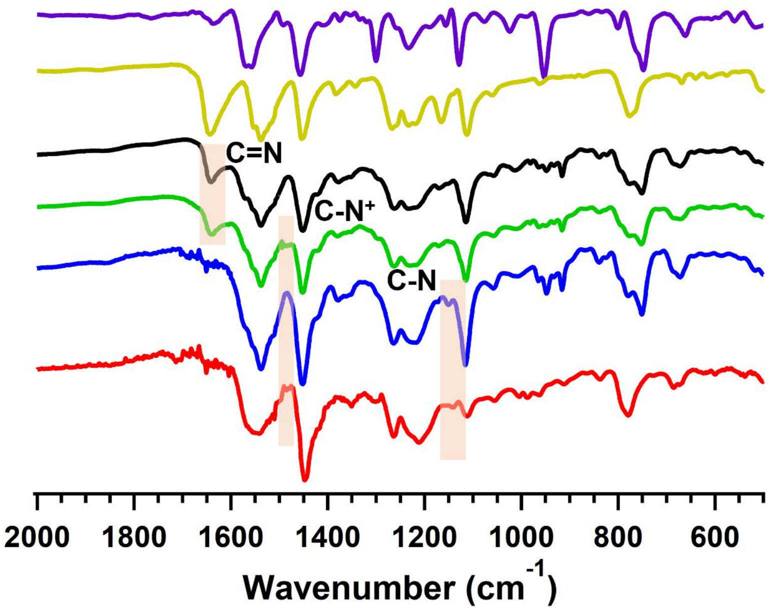

Supplementary Figure 1. FT IR spectra of CoTAPP-PATA-COF (black), N^+-COF (green), NH-COF (blue), N^+-NH-COF (red), PATA (yellow) and CoTAPP (purple).

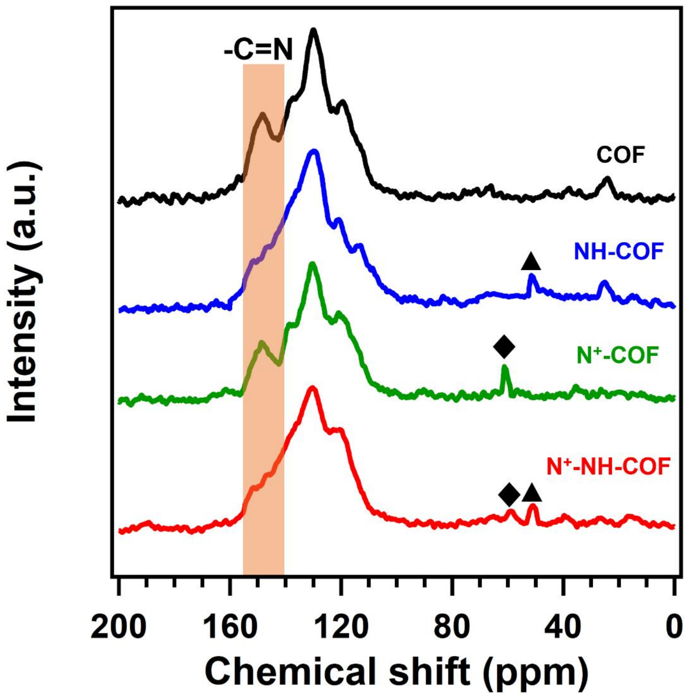

Supplementary Figure 2. The solid-state ^{13}C NMR spectra of CoTAPP-PATA-COF (black), N^{+}-COF (green), NH-COF (blue), N^{+}-NH-COF (red), PATA (yellow) and CoTAPP (purple).

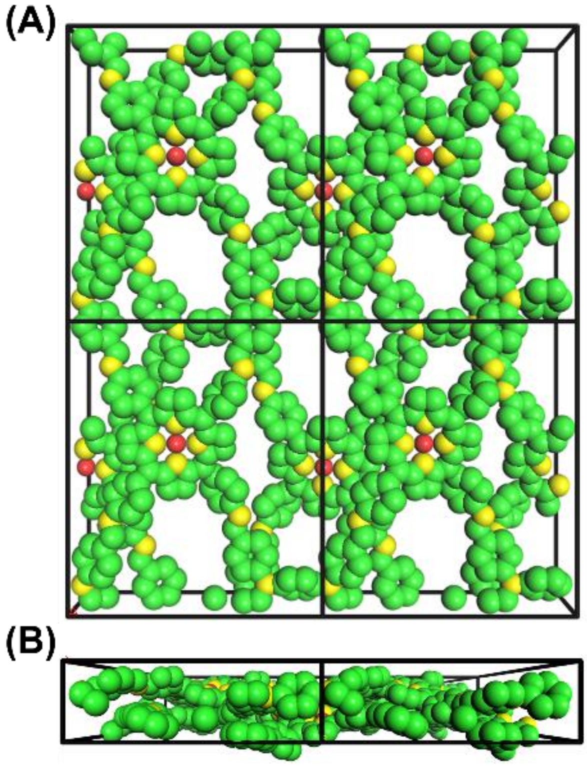

Supplementary Figure 3. Staggered-AB model of CoTAPP-PATA-COF for (A) top view and (B) side view.

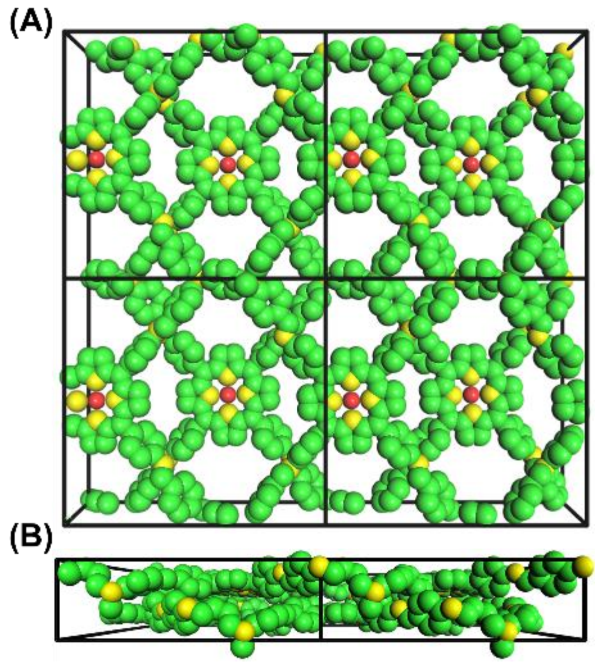

Supplementary Figure 4. Staggered-AB model of N^+-COF for (A) top view and (B) side view.

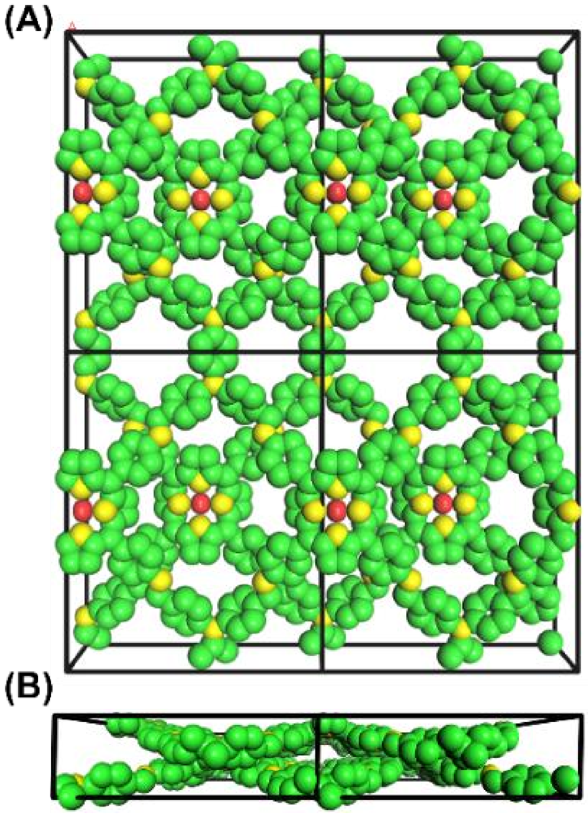

Supplementary Figure 5. Staggered-AB model of NH-COF for (A) top view and (B) side view.

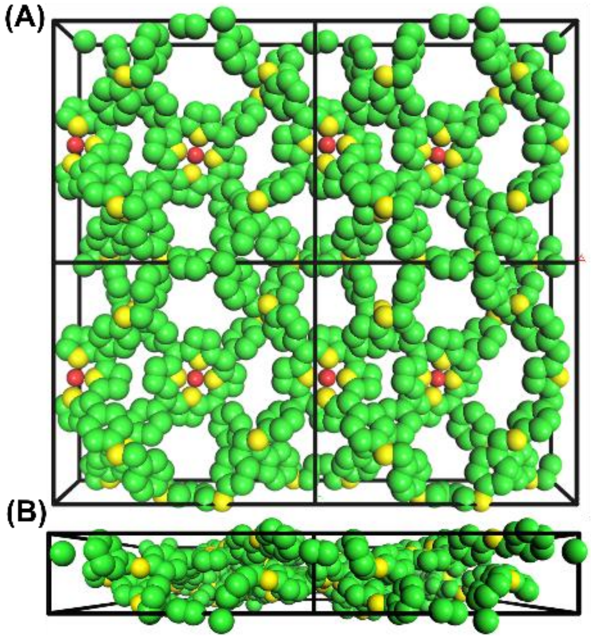

Supplementary Figure 6. Staggered-AB model of N^+-NH-COF for (A) top view and (B) side view.

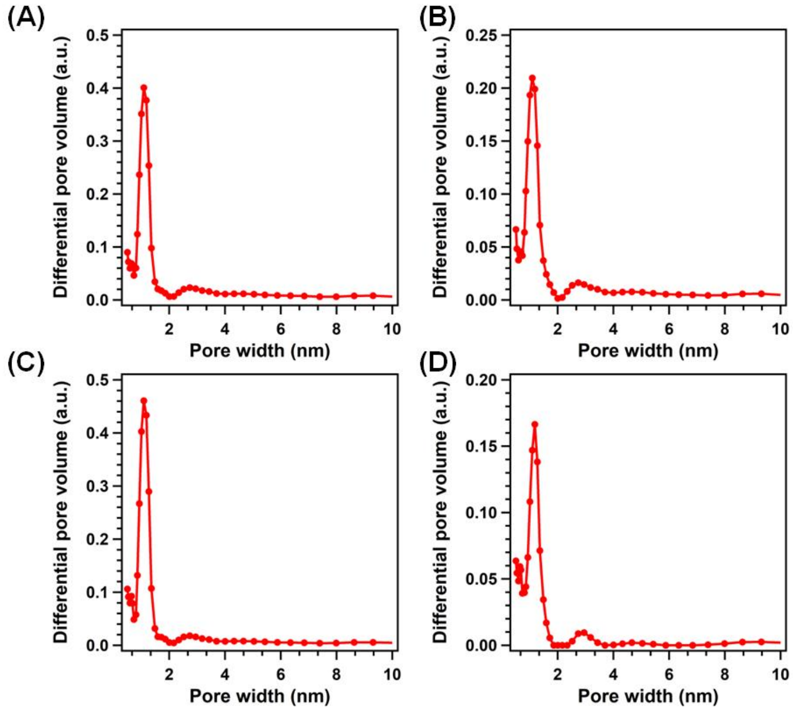

Supplementary Figure 7. The pore distribution curves of (A) CoTAPP-PATA-COF, (B) N^+-COF, (C) NH-COF and (D) N^+-NH-COF.

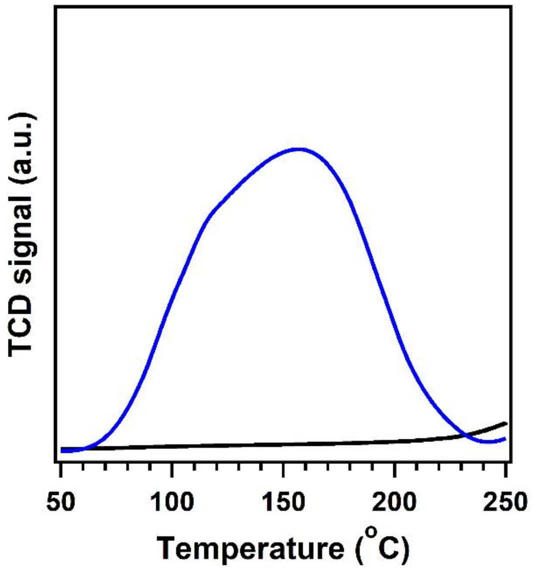

Supplementary Figure 8. The CO_2-TPD curves of CoTAPP-PATA-COF (black) and NH-COF (blue).

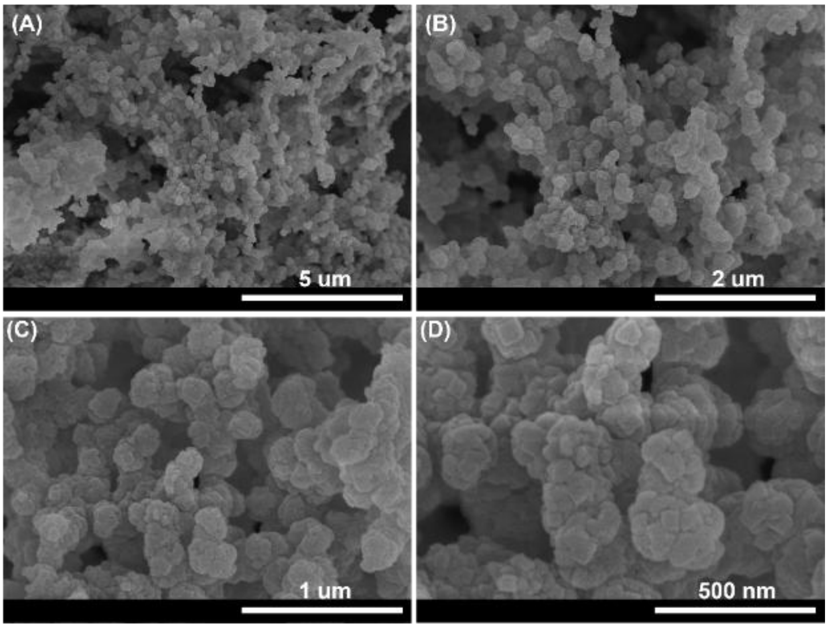

Supplementary Figure 9. FE-SEM images of CoTAPP-PATA-COF (scale bar: (A) 5 μm, (B) 2 μm, (C) 1 μm and (D) 500 nm).

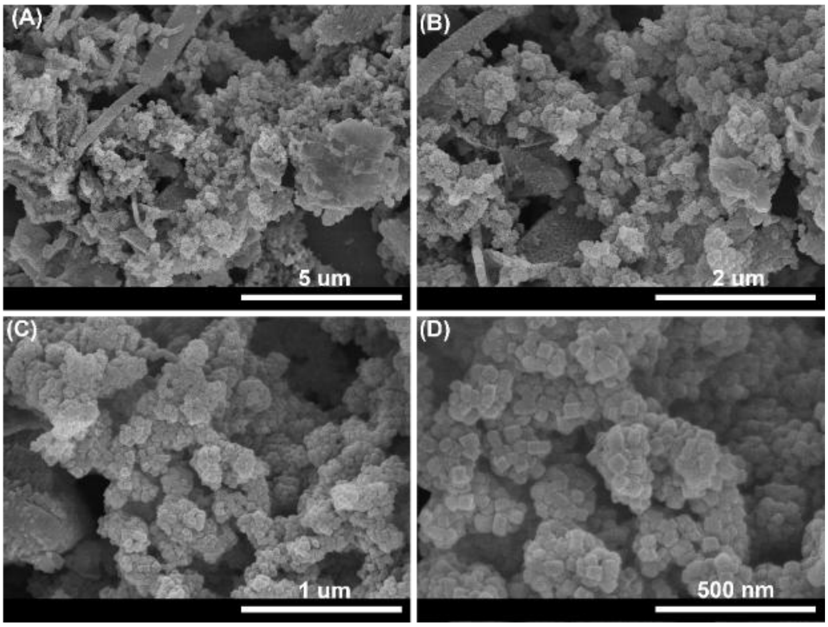

Supplementary Figure 10. FE-SEM images of N^+-COF (scale bar: (A) 5 μm, (B) 2 μm, (C) 1 μm and (D) 500 nm).

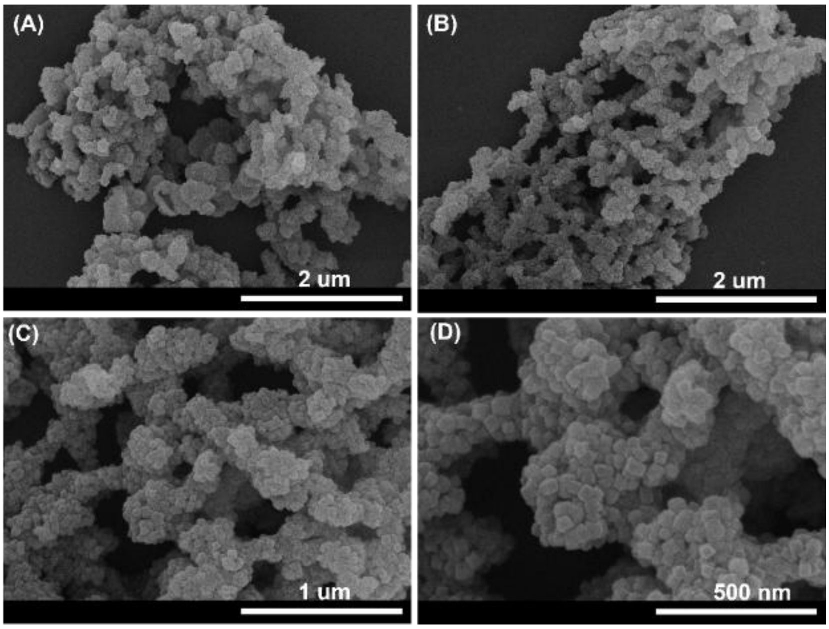

Supplementary Figure 11. FE-SEM images of NH-COF (scale bar: (A) 2 μm, (B) 2 μm, (C) 1 μm and (D) 500 nm).

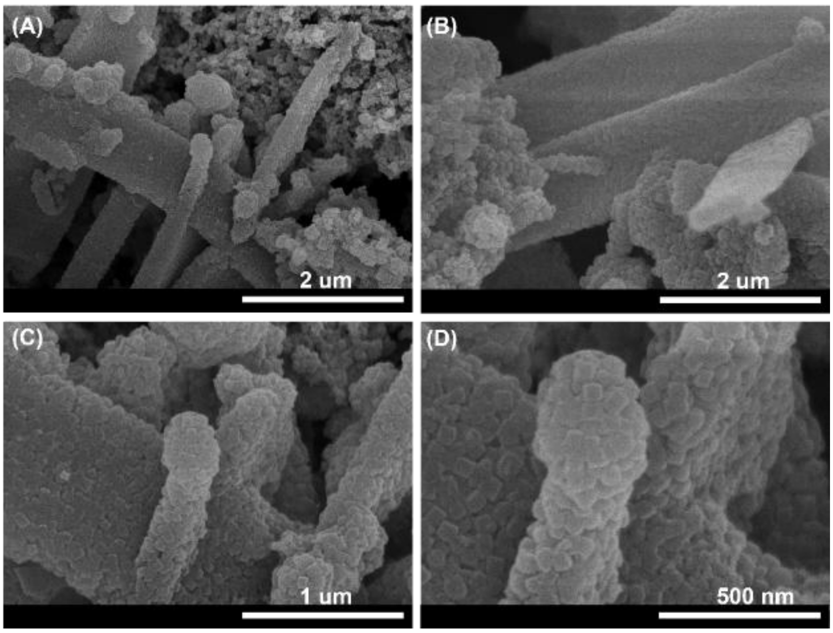

Supplementary Figure 12. FE-SEM images of N^+-NH-COF (scale bar: (A) 2 μm, (B) 2 μm, (C) 1 μm and (D) 500 nm).

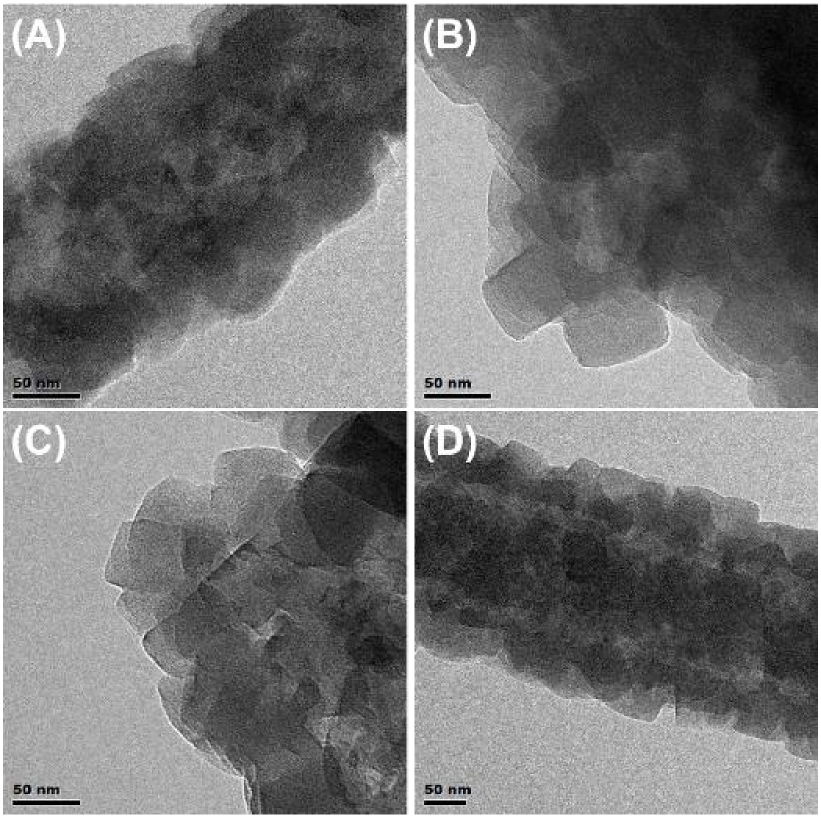

Supplementary Figure 13. (A-D) The TEM images of CoTAPP-PATA-COF at different area.

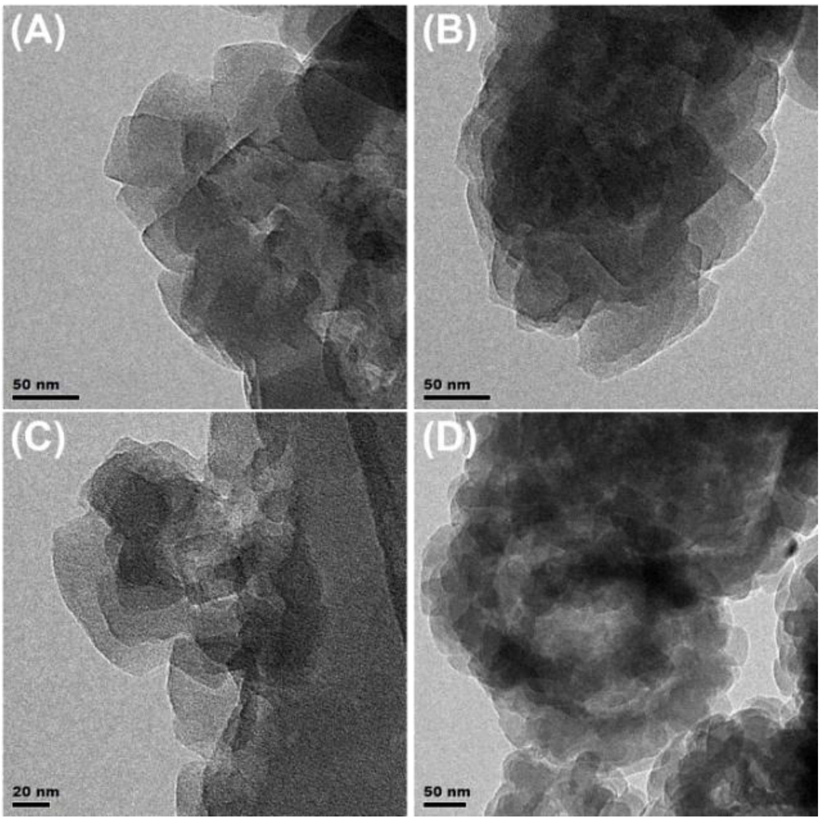

Supplementary Figure 14. (A-D) The TEM images of N^+-COF at different area.

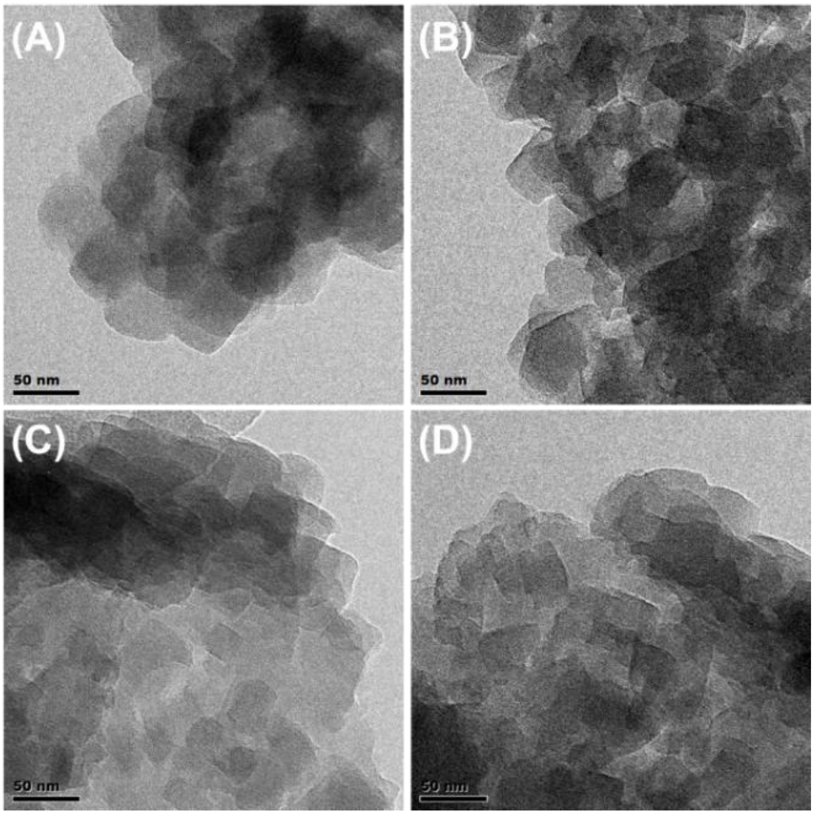

Supplementary Figure 15. (A-D) The TEM images of NH-COF at different area.

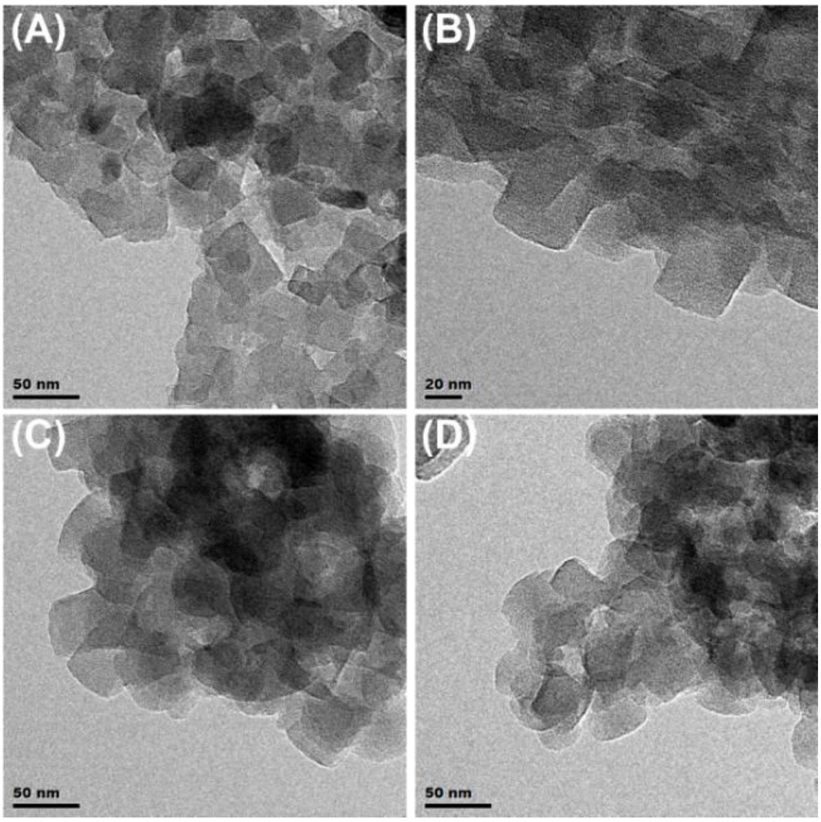

Supplementary Figure 16. (A-D) The TEM images of N^+-NH-COF at different area.

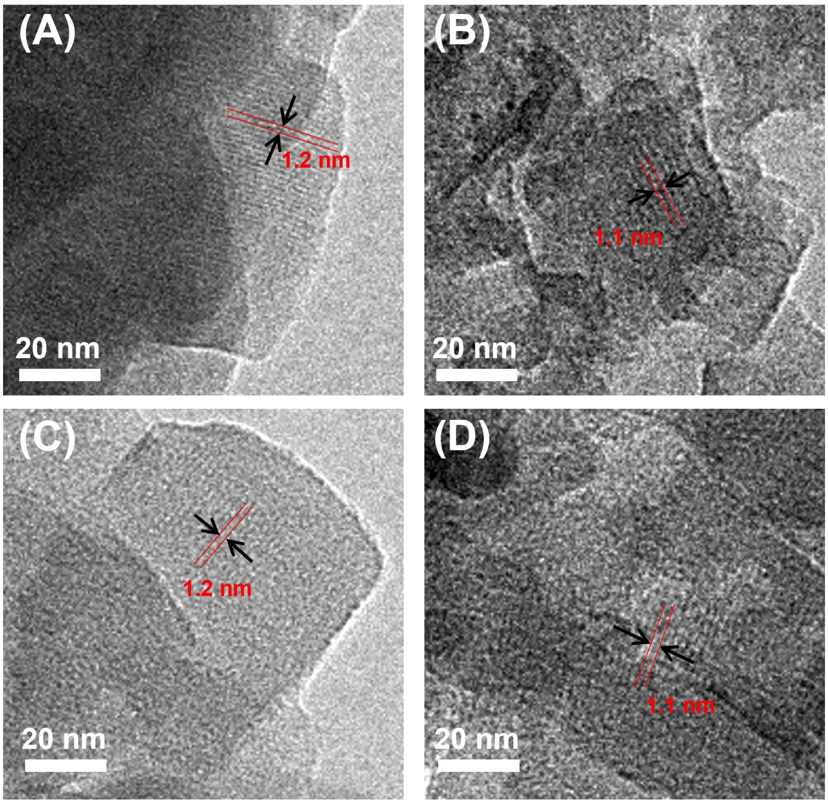

Supplementary Figure 17. The HR-TEM images for (A) CoTAPP-PATA-COF, (B) N^+-COF, (C) NH-COF and (D) N^+-NH-COF.

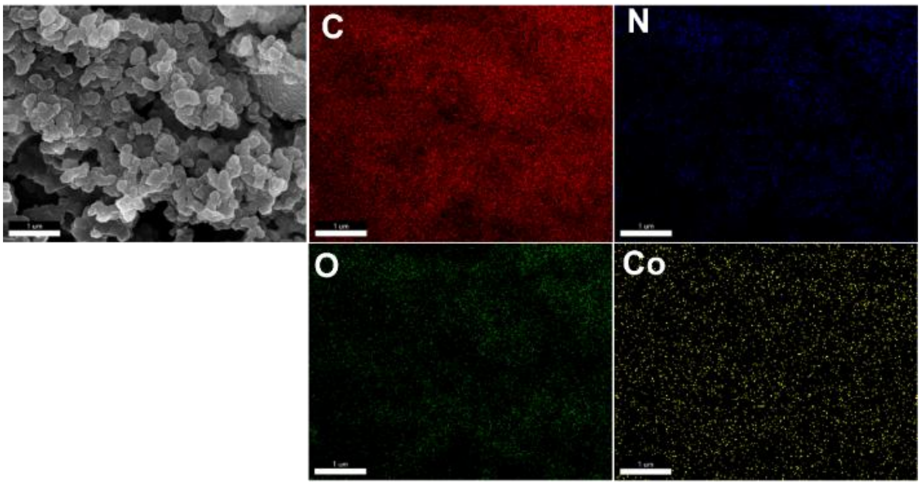

Supplementary Figure 18. EDX-mapping images of CoTAPP-PATA-COF.

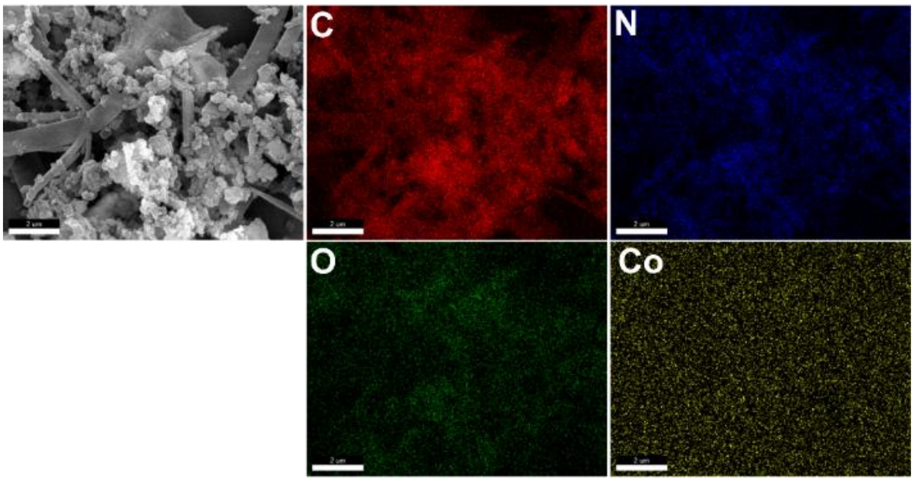

Supplementary Figure 19. EDX-mapping images of N^+-COF.

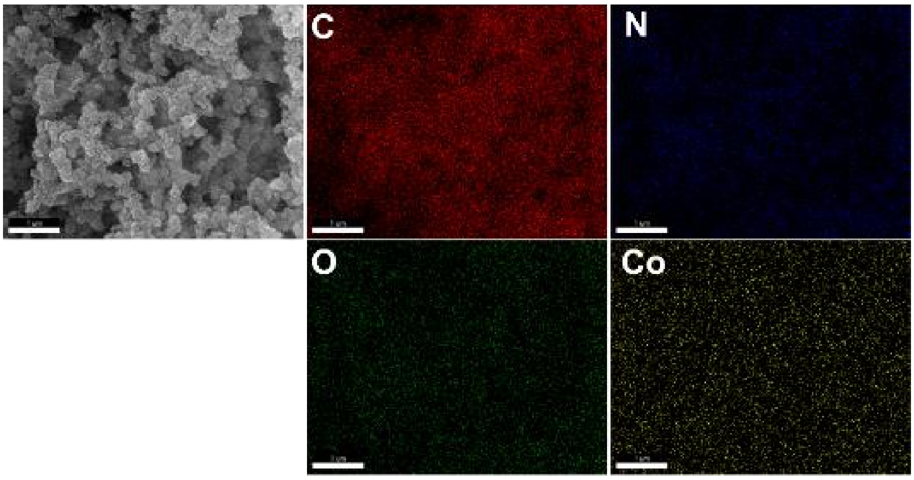

Supplementary Figure 20. EDX-mapping images of NH-COF

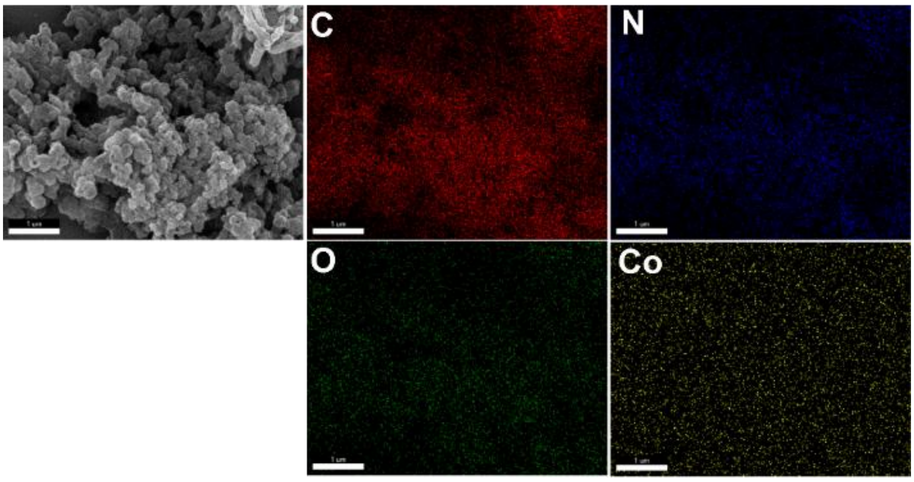

Supplementary Figure 21. EDX-mapping images of N^+-NH-COF

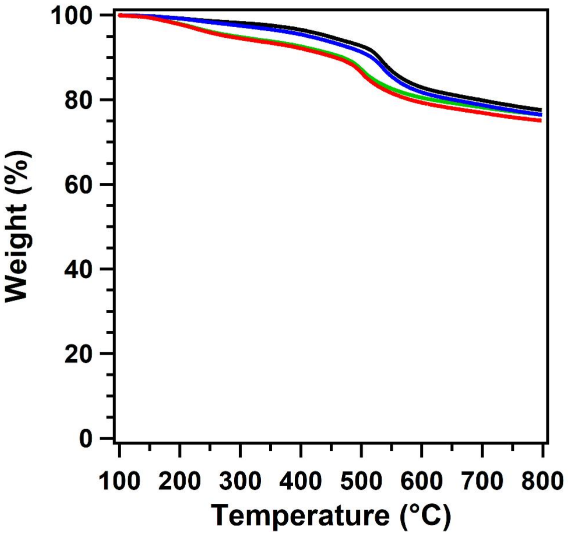

Supplementary Figure 22. TGA curves of CoTAPP-PATA-COF (black), N^+-COF (green), NH-COF (blue) and N^+-NH-COF (red).

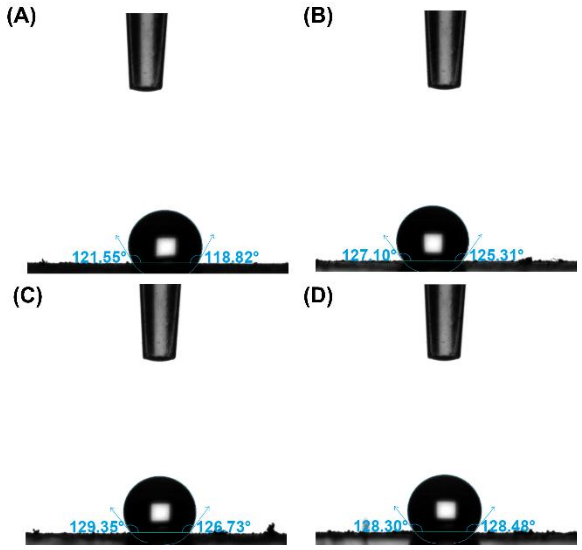

Supplementary Figure 23. The contact angles of water for (A) CoTAPP-PATA-COF, (B) N^+-COF, (C) NH-COF and (D) N^+-NH-COF.

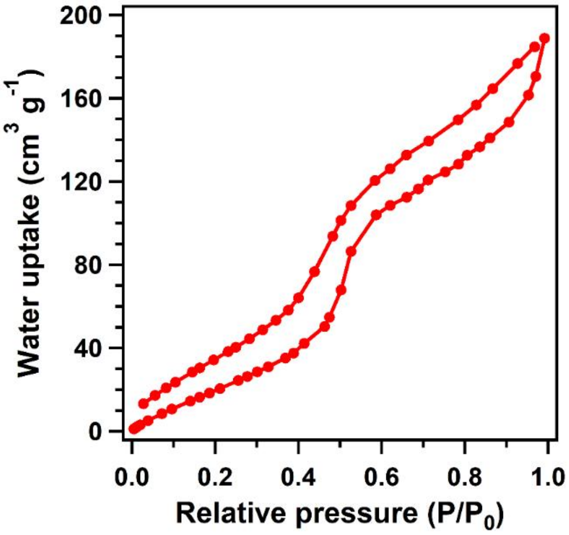

Supplementary Figure 24. The water uptakes of N^+-NH-COF at 298 K.

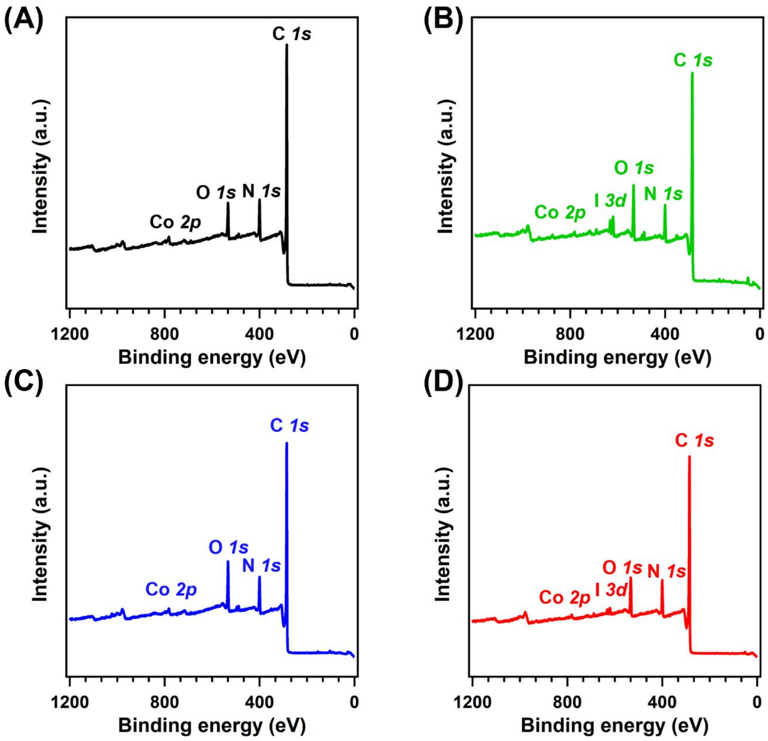

Supplementary Figure 25. The XPS spectra of (A) CoTAPP-PATA-COF, (B) N^+-COF, (C) NH-COF and (D) N^+-NH-COF.

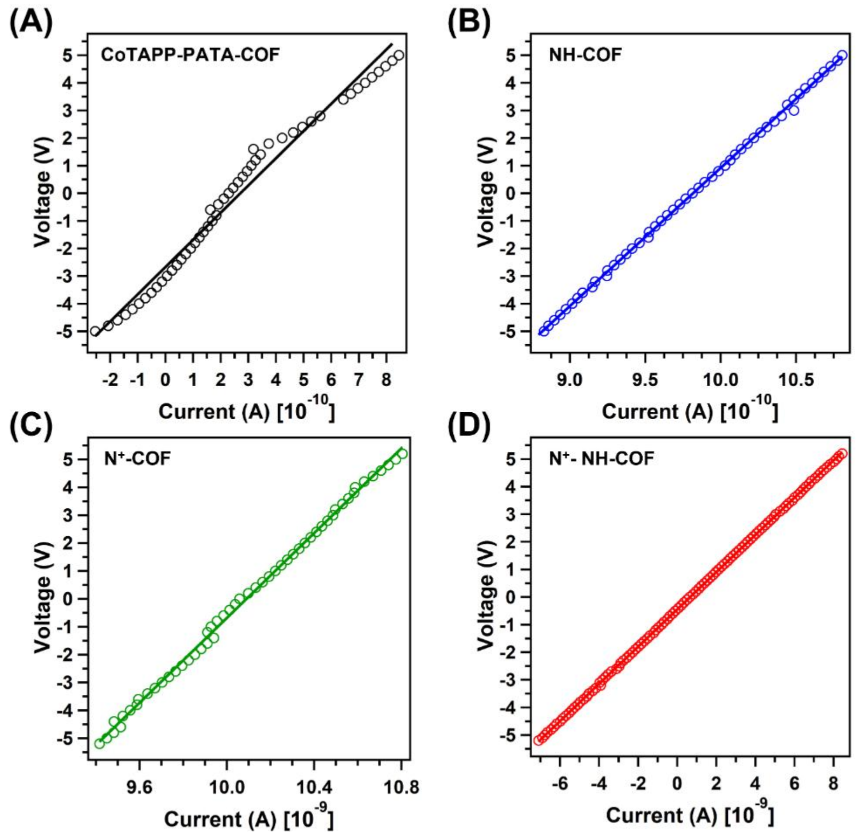

Supplementary Figure 26. The I-V curves of (A) CoTAPP-PATA-COF, (B) NH-COF, (C) N^+-COF and (D) N^+-NH-COF by the four-probe measurement at 298 K.

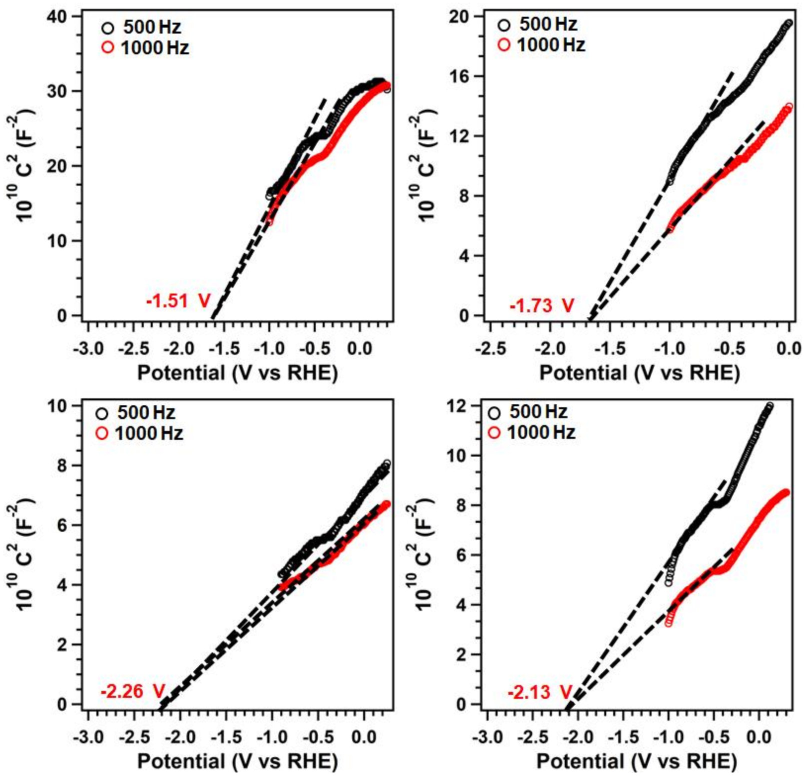

Supplementary Figure 27. Mott–Schottky plots of (A) CoTAPP-PATA-COF, (B) N^+-COF, (C) NH-COF and (D) N^+-NH-COF from 500 Hz and 1000 Hz.

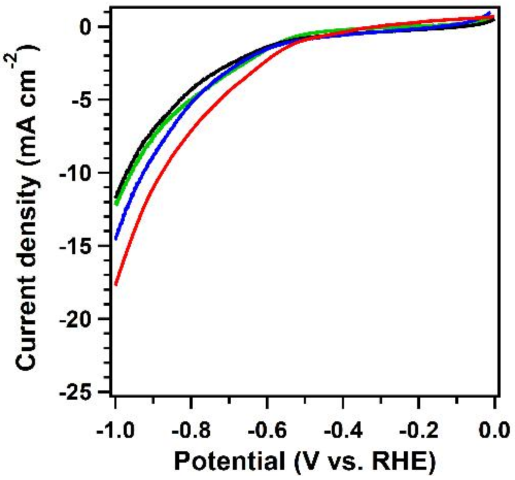

Supplementary Figure 28. The LSV curves of (A) CoTAPP-PATA-COF, (B) N^+-COF, (C) NH-COF and (D) N^+-NH-COF in 0.5 M $KHCO_3$ under N_2 atmosphere.

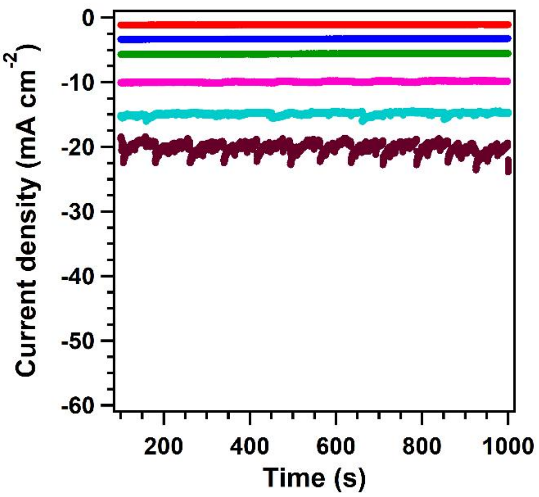

Supplementary Figure 29. Chronoamperometric responses of CoTAPP-PATA-COF at –0.5 (red), –0.6 (blue), –0.7 (green), –0.8 (pink), –0.9 (cyan), and –1.0 V (brown) (vs. RHE).

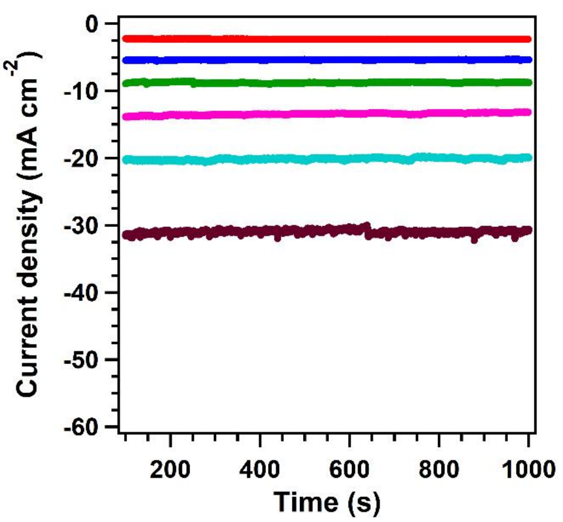

Supplementary Figure 30. Chronoamperometric responses of N^+-COF at –0.5 (red), –0.6 (blue), –0.7 (green), –0.8 (pink), –0.9 (cyan), and –1.0 V (brown) (vs. RHE).

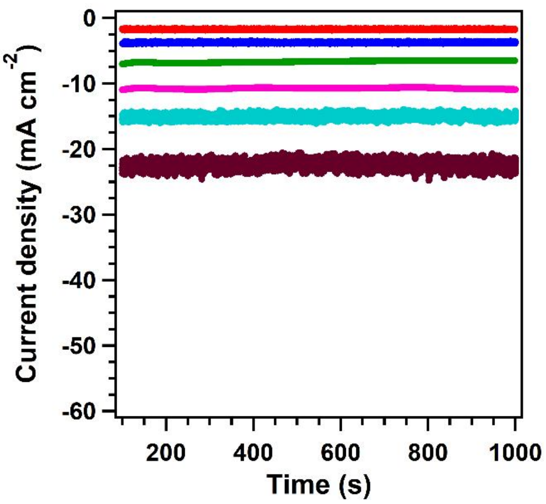

Supplementary Figure 31. Chronoamperometric responses of NH-COF at –0.5 (red), –0.6 (blue), –0.7 (green), –0.8 (pink), –0.9 (cyan), and –1.0 V (brown) (vs. RHE).

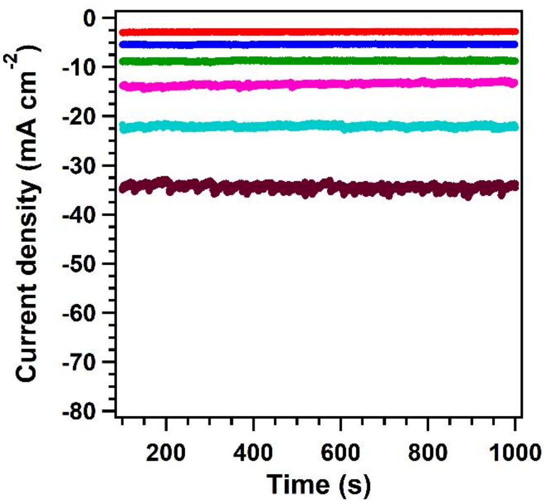

Supplementary Figure 32. Chronoamperometric responses of N^+-NH-COF at –0.5 (red), –0.6 (blue), –0.7 (green), –0.8 (pink), –0.9 (cyan), and –1.0 V (brown) (vs. RHE).

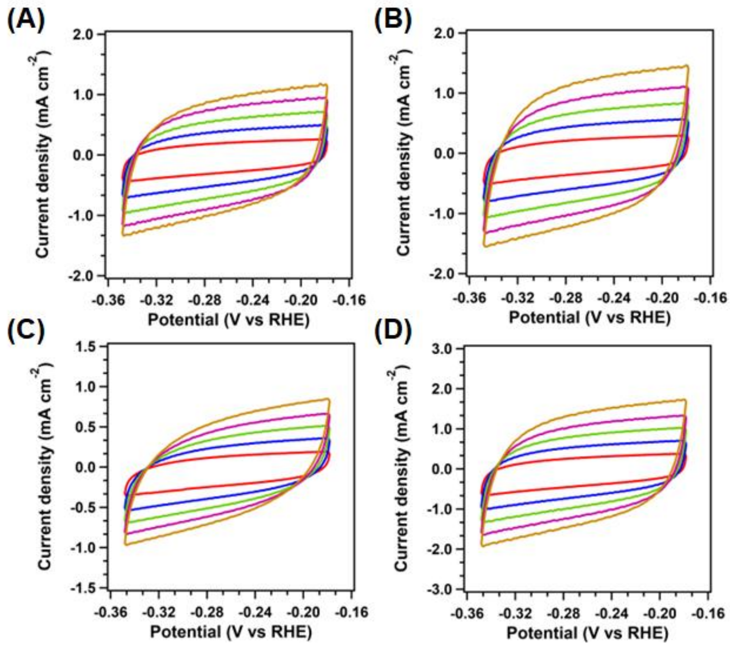

Supplementary Figure 33. The CV curves from 10 mV s^{-1} to 50 mV s^{-1} for (A) CoTAPP-PATA-COF, (B) N^{+}-COF, (C) NH-COF and (D) N^{+}-NH-COF.

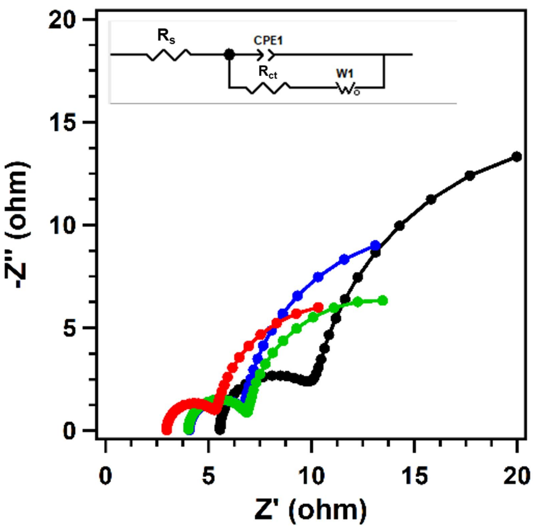

Supplementary Figure 34. EIS spectra of CoTAPP-PATA-COF (black), N^+-COF (green), NH-COF (blue) and N^+-NH-COF (red).

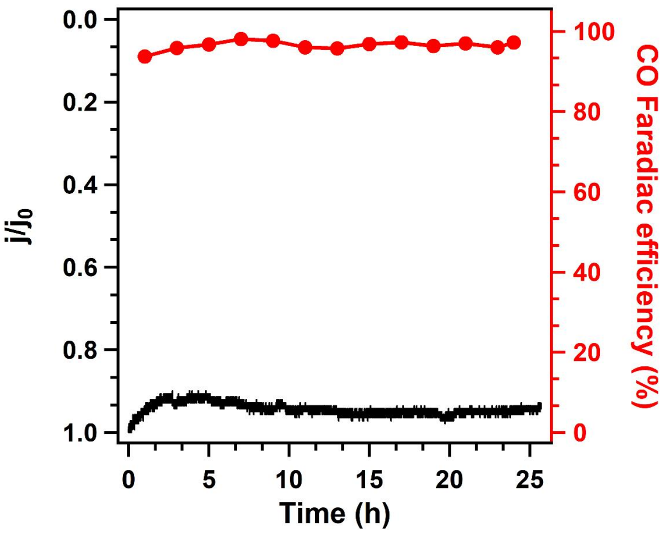

Supplementary Figure 35. Chronoamperometry test for CO_2RR of N^+-NH-COF at a potential of -0.8 V in 0.5 M $KHCO_3$ under CO_2 atmosphere.

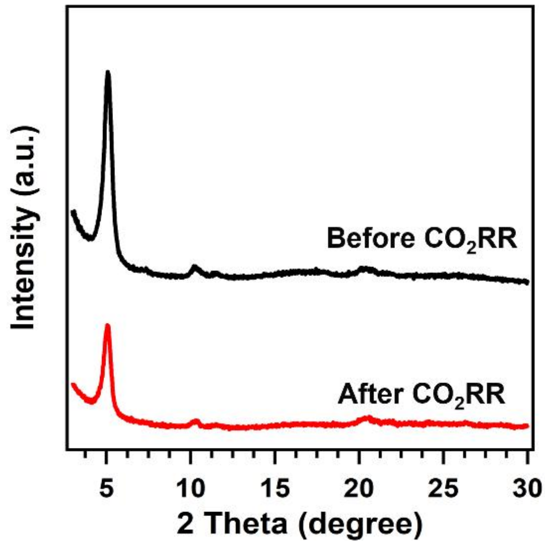

Supplementary Figure 36. The PXRD patterns of N^+-NH-COF before (black) or after (red) the chronoamperometry tests.

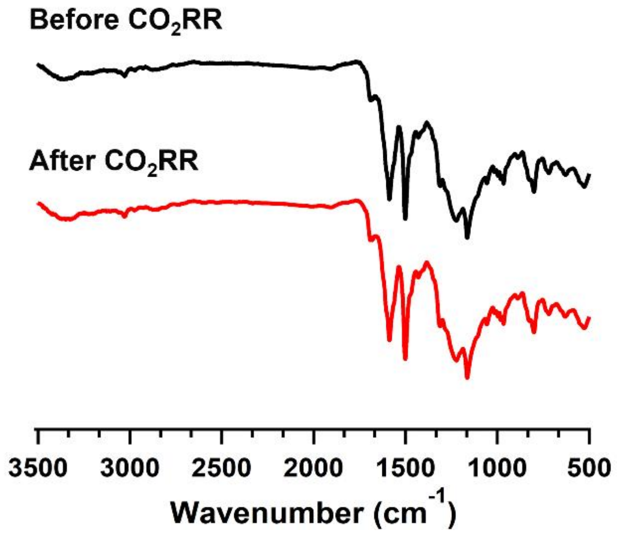

Supplementary Figure 37. The FT IR spectra of N^+-NH-COF before (black) or after (red) the chronoamperometry tests.

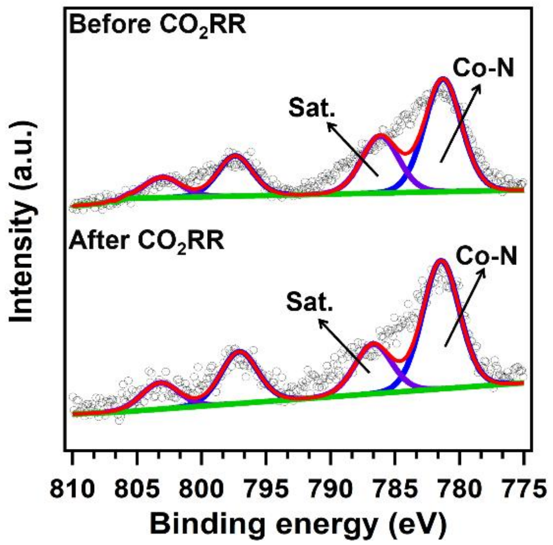

Supplementary Figure 38. XPS for Co *2p* of N^{+}-NH-COF before or after the chronoamperometry tests.

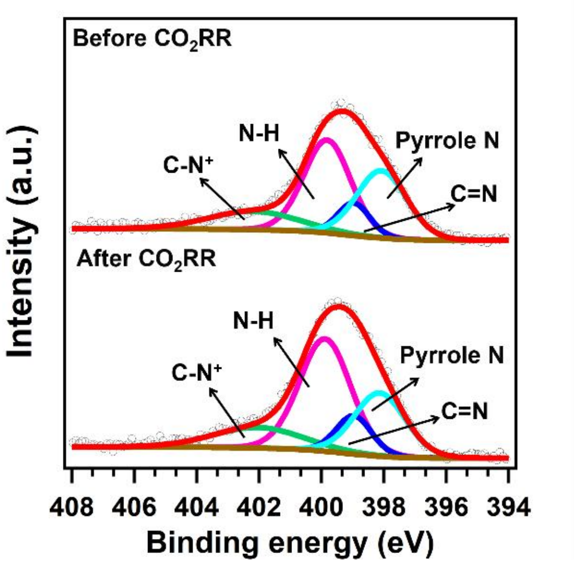

Supplementary Figure 39. XPS for N *1s* of N^+-NH-COF before or after the chronoamperometry tests.

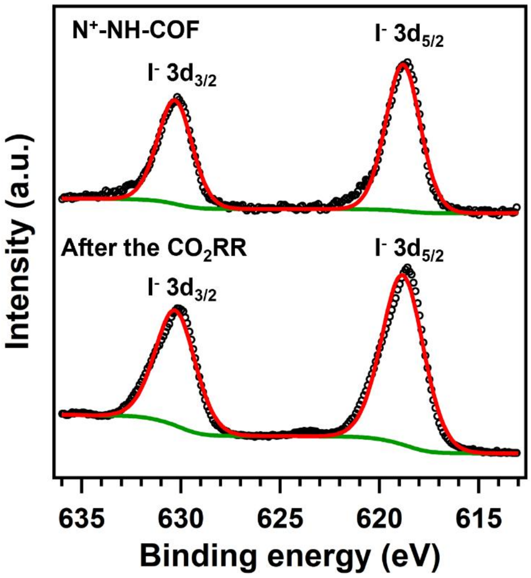

Supplementary Figure 40. The XPS spectra for I^- of N^+-NH-COF before and after the chronoamperometry measurement.

The introduced counterions makes lower potential energy surface, thus, we further considered the exist formation of I^- after the CO_2RR. We tested the XPS spectra before and after the CO_2RR test of the N^+-NH-COF (Supplementary Fig. 40). The fine I^- *3d* XPS spectrum of N^+-NH-COF verifies only free iodide ions is observed (*$3d_{5/2}$* at 630.5 eV, *$3d_{3/2}$* at 619.0 eV). After the electrolysis, I^- shows no significant change compared with the corresponding freshly prepared material.

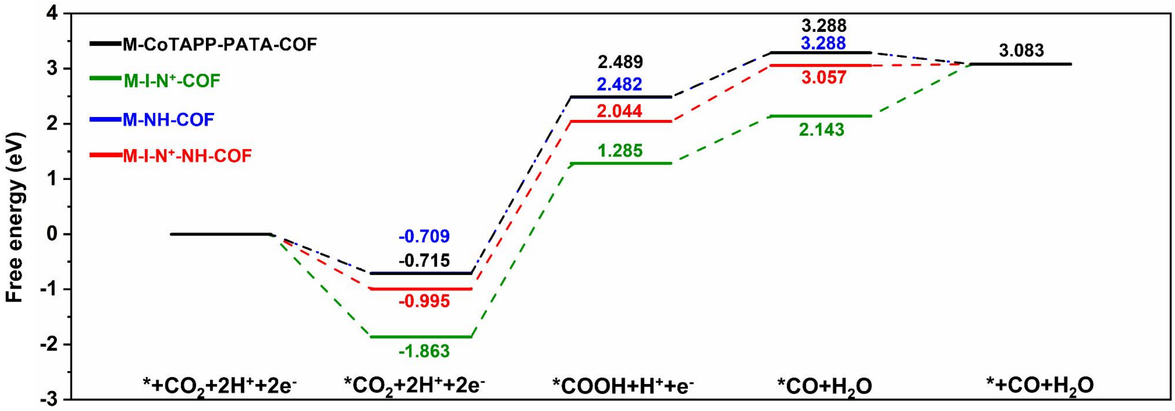

Supplementary Figure 41. The calculated free energy (*G*) diagram of M-CoTAPP-PATA-COF, M-I-N+-COF, M-NH-COF and M-I-N+-NH-COF catalyzing CO_2RR (M-Model).

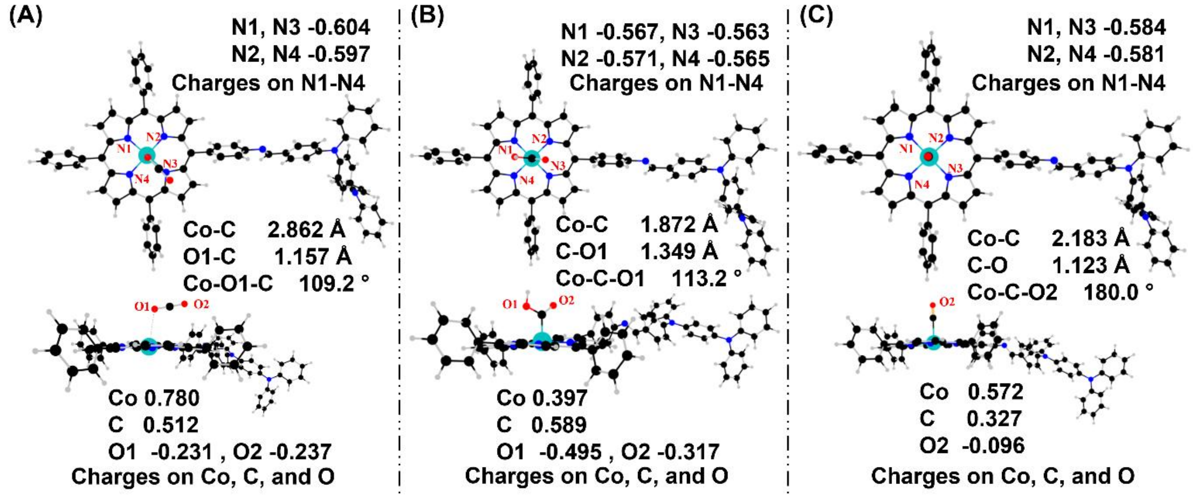

Supplementary Figure 42. The optimized geometrical structures of stationary points along the reaction pathway of CoTAPP-PATA-COF catalyzing CO_2RR reaction, (A) CO_2*, (B) COOH*, (C) CO*, along with the main geometrical parameters and the Mulliken charges on main atoms.

Supplementary tables

Supplementary Table 1. Atomistic coordinates of AA-COF for CoTAPP-PATA-COF optimized by using CASTEP- method.

Space group: *PM6*;

a = 3.7656 Å, b = 23.8027 Å, c = 25.2895 Å;

α = β =γ = 90 °.

Atom	*x/a*	*y/b*	*z/c*
C1	-0.58804	1.32627	-1.47605
C2	-0.5774	1.32627	-1.52783
C3	-0.49361	1.38367	-1.54397
N4	-0.46541	1.41801	-1.50145
C5	-0.51118	1.38368	-1.45926
C6	-0.33715	1.52857	-1.34119
C7	-0.41571	1.45506	-1.39228
C8	-0.47463	1.39902	-1.40633
C9	-0.27038	1.47142	-1.66025
C10	-0.36965	1.54496	-1.60997
C11	-0.43508	1.39899	-1.5965
C12	-0.50608	1.64474	-1.36356
C13	-0.45142	1.35524	-1.63951
C14	-0.35765	1.69869	-1.36908
C15	-0.37465	1.73777	-1.32771
C16	-0.53762	1.72342	-1.27986
C17	-0.69085	1.67003	-1.27444
C18	-0.67755	1.63162	-1.31584
C19	-0.6022	1.63144	-1.68872
C20	-0.60434	1.6701	-1.72998
C21	-0.46159	1.72394	-1.72283
C22	-0.32171	1.73867	-1.67351

C23	-0.31349	1.69921	-1.6324
N24	-0.45319	1.23672	-1.76566
C25	-0.70493	0.9707	-2.04229
C26	-0.54135	1.0596	-2.00135
C27	-0.37754	1.02933	-1.96041
N28	-0.54109	1.12061	-2.00155
C29	-0.49424	1.15207	-1.95271
C30	-0.58916	1.15113	-2.05084
C31	-0.34692	1.20627	-1.95386
C32	-0.32244	1.23862	-1.90816
C33	-0.43829	1.21715	-1.8599
C34	-0.57867	1.1627	-1.85748
C35	-0.60825	1.13056	-1.9037
C36	-0.45407	1.13038	-2.09894
C37	-0.4844	1.1618	-2.14557
C38	-0.64878	1.21461	-2.14452
C39	-0.78728	1.2351	-2.09719
C40	-0.7595	1.20361	-2.05099
C41	-0.66561	1.25121	-2.1909
C42	-0.42895	1.25443	-1.81398
N43	-0.53008	1.23794	-2.23635
H44	-0.64927	1.28577	-1.45378
H45	-0.6295	1.28578	-1.55062
H46	-0.27864	1.55252	-1.30314
H47	-0.19643	1.44747	-1.6977
H48	-0.22132	1.7111	-1.4076
H49	-0.25542	1.78144	-1.33297
H50	-0.82756	1.65784	-1.23589
H51	-0.80733	1.58861	-1.31136

H52	-0.88914	1.61836	-1.68129
H53	-0.45864	1.59367	-1.70637
H54	-0.72269	1.65806	-1.76989
H55	-0.21409	1.78284	-1.66669
H56	-0.83899	0.94675	-2.07584
H57	-0.24219	1.05264	-1.92658
H58	-0.24489	1.22447	-1.99269
H59	-0.20725	1.28286	-1.91013
H60	-0.66862	1.1446	-1.81795
H61	-0.72572	1.08646	-1.90189
H62	-0.31828	1.0876	-2.10023
H63	-0.37543	1.1444	-2.18431
H64	-0.92402	1.27782	-2.0962
H65	-0.87605	1.22033	-2.01248
H66	-0.80309	1.29378	-2.18814
H67	-0.39859	1.30125	-1.82234
N68	-0.44927	1.5	-1.42466
N69	-0.41627	1.5	-1.57795
Co70	-0.45013	1.5	-1.50139

Supplementary Table 2. Atomistic coordinates of AB-COF for N^+-COF optimized by using CASTEP- method.

Space group: *P1*;

a = 24.3812 Å, b = 23.9803 Å, c = 5.6341 Å;

α = β =γ = 90˚.

Atom	*x/a*	*y/b*	*z/c*
C1	0.21211	-0.18737	0.5264
C2	0.20595	-0.18737	0.47433
C3	0.22942	-0.12916	0.45763
N4	0.24653	-0.09451	0.49998
C5	0.23923	-0.12914	0.54255
C6	0.30754	0.0159	0.66202
C7	0.27581	-0.05725	0.61005
C8	0.2557	-0.11319	0.59549
C9	0.28426	-0.04103	0.33766
C10	0.25794	0.03222	0.38979
C11	0.23455	-0.11325	0.40445
C12	0.24993	0.1316	0.63808
C13	0.20457	-0.15651	0.36213
C14	0.36901	0.17839	0.63616
C15	0.37075	0.21698	0.67786
C16	0.25627	0.20915	0.72241
C17	0.13621	0.16253	0.7243
C18	0.13195	0.1245	0.68232
C19	0.10329	0.11914	0.3096
C20	0.15166	0.15714	0.26644
C21	0.26544	0.20616	0.27404
C22	0.34086	0.21895	0.32805
C23	0.29476	0.17837	0.3593

N24	0.30476	-0.2662	0.23296
C25	0.15622	0.45826	0.96587
C26	0.27516	-0.45336	1.00003
C27	0.39546	-0.48322	0.0341
N28	0.27462	-0.39281	0.99874
C29	0.29125	-0.35899	0.04651
C30	0.23851	-0.36526	0.94869
C31	0.31744	-0.30078	0.04231
C32	0.31434	-0.26657	0.08705
C33	0.28915	-0.28983	0.13728
C34	0.26889	-0.34792	0.14273
C35	0.26904	-0.38225	0.09752
C36	0.35318	-0.37579	0.9044
C37	0.32671	-0.34566	0.85726
C38	0.18518	-0.30485	0.85416
C39	0.06778	-0.29557	0.89773
C40	0.09336	-0.32568	0.94458
C41	0.16578	-0.26785	0.80812
C42	0.26727	-0.25162	0.18217
N43	0.26833	-0.27294	0.76585
C44	0.21182	0.16223	0.52643
C45	0.20557	0.16224	0.47437
C46	0.22912	0.10403	0.45765
N47	0.24638	0.06937	0.5
C48	0.23909	0.104	0.54257
C49	0.30755	-0.04105	0.66202
C50	0.27579	0.0321	0.61006
C51	0.25564	0.08804	0.5955
C52	0.28416	0.01594	0.33766

C53	0.2581	-0.05733	0.38979
C54	0.23423	0.08813	0.40447
C55	0.24997	-0.15676	0.63807
C56	0.20408	0.1314	0.36216
C57	0.36906	-0.20355	0.63614
C58	0.37081	-0.24214	0.67785
C59	0.25635	-0.2343	0.7224
C60	0.13628	-0.18768	0.72429
C61	0.132	-0.14966	0.6823
C62	0.10378	-0.14427	0.30956
C63	0.15215	-0.18229	0.26642
C64	0.26595	-0.2313	0.27402
C65	0.34138	-0.24407	0.32804
C66	0.29526	-0.20349	0.35928
N67	0.30424	0.24105	0.23297
C68	0.15628	-0.48339	0.96587
C69	0.27505	0.42822	1.00004
C70	0.3954	0.45806	0.0341
N71	0.27441	0.36766	0.99875
C72	0.29093	0.33384	0.04651
C73	0.23831	0.34013	0.94869
C74	0.3169	0.27562	0.04231
C75	0.31371	0.2414	0.08705
C76	0.28866	0.26468	0.13729
C77	0.26862	0.32277	0.14274
C78	0.26887	0.3571	0.09752
C79	0.3531	0.35063	0.90442
C80	0.32664	0.3205	0.85728
C81	0.18501	0.27972	0.85416

C82	0.0675	0.27047	0.89771
C83	0.09307	0.30058	0.94457
C84	0.16562	0.24272	0.80811
C85	0.26673	0.22647	0.18218
N86	0.26822	0.2478	0.76585
N87	0.26163	-0.01257	0.57725
N88	0.2499	-0.01256	0.42272
Co89	0.25053	-0.01257	0.49999

Supplementary Table 3. Atomistic coordinates of AA-COF for NH-COF optimized by using CASTEP- method.

Space group: *PM;*

a = 3.7888 Å, b = 26.9934 Å, c = 22.6581 Å;

$\alpha = \beta = \gamma = 90$ °.

Atom	*x/a*	*y/b*	*z/c*
N1	-0.48147	3.26146	-1.77951
C2	-0.4555	2.97415	-2.03701
C3	-0.61987	3.05249	-1.99143
C4	-0.78396	3.02585	-1.94583
C5	-0.63516	3.13316	-1.93599
C6	-0.60402	3.1333	-2.04677
C7	-0.79565	3.18004	-1.93293
C8	-0.80469	3.20643	-1.87998
C9	-0.65413	3.18626	-1.82903
C10	-0.49757	3.13945	-1.83115
C11	-0.48692	3.1132	-1.88429
C12	-0.74517	3.1131	-2.09888
C13	-0.73259	3.13942	-2.15196
C14	-0.58149	3.18661	-2.1539
C15	-0.44008	3.20708	-2.10219
C16	-0.44994	3.18059	-2.04935
C17	-0.66279	3.21428	-1.77244
N18	-0.61998	3.10626	-1.99142
C19	-0.57983	3.2142	-2.21131
C20	-0.19836	3.34866	-2.46458
C21	-0.20242	3.34865	-2.5228
C22	-0.32973	3.39751	-2.54092
N23	-0.39517	3.42613	-2.49337

C24	-0.32343	3.39752	-2.44604
C25	-0.60515	3.52542	-2.31712
C26	-0.47496	3.46024	-2.37156
C27	-0.39995	3.41077	-2.38728
C28	-0.62043	3.47457	-2.6694
C29	-0.48734	3.53977	-2.61516
C30	-0.41372	3.41071	-2.59941
C31	-0.4004	3.62838	-2.34057
C32	-0.4257	3.37127	-2.6457
C33	-0.54841	3.67524	-2.35134
C34	-0.55388	3.71139	-2.3072
C35	-0.41279	3.70108	-2.25137
C36	-0.2642	3.65467	-2.24034
C37	-0.25676	3.61873	-2.2846
C38	-0.28625	3.62012	-2.70222
C39	-0.30403	3.65654	-2.74588
C40	-0.45961	3.70232	-2.73372
C41	-0.59687	3.71151	-2.67735
C42	-0.58051	3.67495	-2.63382
N43	-0.42411	3.26328	-2.20486
Co44	0.60007	0.49875	1.50849
H45	-0.35179	3.27065	-1.82205
H46	-0.32093	2.95303	-2.07432
H47	-0.91936	3.04645	-1.90819
H48	-0.92056	3.19697	-1.97402
H49	-0.93492	3.24448	-1.87825
H50	-0.37838	3.12263	-1.78952
H51	-0.35695	3.07514	-1.88608
H52	-0.87188	3.0748	-2.09817

H53	-0.84593	3.1224	-2.19392
H54	-0.31624	3.24556	-2.10318
H55	-0.33201	3.19727	-2.00755
H56	-0.9487	3.22129	-1.75917
H57	-0.52628	3.19163	-1.73663
H58	-0.86262	3.21818	-2.22783
H59	-0.41946	3.19261	-2.24523
H60	-0.10768	3.31429	-2.43958
H61	-0.11549	3.31425	-2.54811
H62	-0.7019	3.54654	-2.27666
H63	-0.71932	3.45345	-2.70972
H64	-0.6657	3.68409	-2.39646
H65	-0.67325	3.74935	-2.31659
H66	-0.14838	3.64603	-2.19506
H67	-0.13303	3.58099	-2.27553
H68	-0.41905	3.58545	-2.72044
H69	0.00818	3.61198	-2.69854
H70	-0.19132	3.64896	-2.79165
H71	-0.72225	3.7489	-2.66694
H72	-0.3062	3.27457	-2.16208
N73	-0.39993	3.5	-2.40546
N74	-0.41006	3.5	-2.5814

Supplementary Table 4. Atomistic coordinates of N^+-NH-COF optimized by using CASTEP-method.

Space group: *PM;*

a = 26.0455 Å, b = 3.4873 Å, c = 24.3666 Å;

$\alpha = \beta = \gamma$ = 90 °.

Atom	*x/a*	*y/b*	*z/c*
N1	3.23773	-1.77646	-0.14242
N2	3.7699	-1.75551	-0.91501
C3	2.92803	-2.0427	-0.34928
C4	2.983	-2.03996	-0.23091
C5	2.99907	-1.99237	-0.08645
C6	2.95733	-1.94788	-0.06558
C7	2.90234	-1.9508	-0.18356
C8	2.88634	-1.99804	-0.32951
C9	3.09412	-1.94202	-0.04904
C10	3.08888	-2.04311	0.02466
C11	3.15239	-1.93497	0.04349
C12	3.18949	-1.89183	-0.0456
C13	3.16881	-1.85397	-0.2258
C14	3.11115	-1.85959	-0.31665
C15	3.07436	-1.90304	-0.22961
C16	3.08428	-2.08506	0.21083
C17	3.11375	-2.13724	0.18977
C18	3.14798	-2.14923	-0.01865
C19	3.15199	-2.10865	-0.2079
C20	3.1225	-2.05651	-0.18744
C21	3.20843	-1.80965	-0.33434
N22	3.0566	-1.98878	0.04119
C23	3.18025	-2.20508	-0.0307
C24	3.31183	-2.46329	-0.31482
C25	3.3141	-2.51903	-0.29755
C26	3.37295	-2.53564	-0.38519
N27	3.40562	-2.48986	-0.45357
C28	3.36935	-2.44482	-0.41172

C29	3.50837	-2.31445	-0.68646
C30	3.45282	-2.31465	-0.58645
C31	3.43761	-2.37127	-0.55261
N32	3.48198	-2.4066	-0.62371
C33	3.5247	-2.371	-0.70861
C34	3.38275	-2.38781	-0.45677
C35	3.65522	-2.51689	-0.91158
C36	3.65262	-2.46129	-0.93319
C37	3.59598	-2.44393	-0.82475
N38	3.56453	-2.48926	-0.74299
C39	3.6002	-2.53439	-0.78964
C40	3.57907	-2.38726	-0.80711
C41	3.46584	-2.66413	-0.4763
C42	3.52187	-2.66379	-0.57226
C43	3.53459	-2.60748	-0.62674
N44	3.48908	-2.57242	-0.56632
C45	3.44719	-2.60793	-0.47489
C46	3.39123	-2.59202	-0.39245
C47	3.58823	-2.59085	-0.73138
C48	3.62135	-2.3427	-0.89111
C49	3.33686	-2.34325	-0.40535
C50	3.3488	-2.63721	-0.31895
C51	3.63479	-2.63439	-0.78043
C52	3.67539	-2.3353	-0.76696
C53	3.71532	-2.29344	-0.84394
C54	3.70162	-2.25839	-1.0458
C55	3.64759	-2.26557	-1.16952
C56	3.6076	-2.30765	-1.09304
C57	3.30831	-2.34022	-0.17539
C58	3.26627	-2.29764	-0.12877
C59	3.25256	-2.25724	-0.31059
C60	3.28066	-2.2602	-0.53977
C61	3.32228	-2.30295	-0.58688
C62	3.69015	-2.63019	-0.66326

C63	3.73438	-2.6705	-0.70938
C64	3.72373	-2.71563	-0.87171
C65	3.66835	-2.72022	-0.98875
C66	3.62411	-2.67969	-0.94368
C67	3.30519	-2.65501	-0.48601
C68	3.26809	-2.70076	-0.42783
C69	3.27411	-2.72905	-0.20116
C70	3.3171	-2.71068	-0.03252
C71	3.35462	-2.66509	-0.09135
N72	3.2117	-2.21192	-0.26408
N73	3.74157	-2.21457	-1.12359
N74	3.82172	-2.00138	-0.44086
C75	3.8143	-2.05347	-0.58826
C76	3.80721	-1.95209	-0.596
C77	3.77319	-2.09652	-0.52377
C78	3.76823	-2.14634	-0.66353
C79	3.8041	-2.15426	-0.87134
C80	3.84502	-2.11241	-0.93797
C81	3.84993	-2.06251	-0.79874
C82	3.84972	-1.91026	-0.65762
C83	3.83487	-1.86359	-0.80512
C84	3.77711	-1.85766	-0.89572
C85	3.73421	-1.89848	-0.8379
C86	3.74906	-1.94519	-0.68995
C87	3.7613	-1.80797	-1.05385
C88	3.80056	-2.20803	-1.01682
C89	0.79478	1.00107	0.76703
C90	1.05415	1.02609	1.30165
Co91	0.50044	1.51306	0.40408
H92	3.23205	-1.78831	0.05465
H93	3.81418	-1.7453	-0.83641
H94	2.916	-2.08164	-0.46498
H95	3.01591	-2.07664	-0.25002
H96	2.96821	-1.90854	0.04947

H97	2.86916	-1.91439	-0.16294
H98	3.16983	-1.96475	0.19328
H99	3.23666	-1.88746	0.02858
H100	3.09397	-1.82863	-0.46242
H101	3.02726	-1.90757	-0.30453
H102	3.05613	-2.07662	0.382
H103	3.1099	-2.17028	0.34334
H104	3.17946	-2.11812	-0.37994
H105	3.12537	-2.02401	-0.34354
H106	3.24353	-1.83126	-0.45378
H107	3.18047	-1.78014	-0.45466
H108	3.14688	-2.24129	-0.00907
H109	3.21404	-2.20743	0.12579
H110	3.26982	-2.43954	-0.25794
H111	3.27415	-2.54371	-0.22459
H112	3.531	-2.27229	-0.73258
H113	3.42902	-2.27265	-0.54842
H114	3.69565	-2.54106	-0.98319
H115	3.69077	-2.43709	-1.02365
H116	3.44458	-2.70608	-0.41723
H117	3.54758	-2.70542	-0.59198
H118	3.68687	-2.36354	-0.60239
H119	3.75929	-2.28762	-0.74261
H120	3.63604	-2.23708	-1.33321
H121	3.5637	-2.31353	-1.1949
H122	3.31933	-2.37259	-0.02514
H123	3.24297	-2.2957	0.05796
H124	3.26965	-2.22766	-0.6893
H125	3.34493	-2.30542	-0.7749
H126	3.69924	-2.5937	-0.52941
H127	3.7794	-2.66665	-0.61442
H128	3.65933	-2.75705	-1.12094
H129	3.5792	-2.68341	-1.03969
H130	3.29985	-2.63214	-0.67045

H131	3.23273	-2.71525	-0.56487
H132	3.3217	-2.73287	0.15382
H133	3.38993	-2.65061	0.04593
H134	3.20439	-2.18101	-0.41633
H135	3.72799	-2.1841	-1.26996
H136	3.7434	-2.09089	-0.35527
H137	3.73488	-2.18061	-0.60813
H138	3.8746	-2.11886	-1.10655
H139	3.88319	-2.02821	-0.85466
H140	3.89722	-1.91422	-0.58665
H141	3.86991	-1.83021	-0.85188
H142	3.687	-1.89375	-0.91135
H143	3.714	-1.97856	-0.6431
H144	3.79094	-1.80753	-1.22456
H145	3.71283	-1.81148	-1.11292
H146	3.81026	-2.24581	-0.89062
H147	3.83477	-2.20687	-1.17197
H148	0.78534	0.95543	0.82785
H149	0.7516	1.02224	0.70628
H150	0.81473	1.02594	0.9278
H151	1.03266	0.98745	1.39293
H152	1.02264	1.06405	1.31304
H153	1.09739	1.03681	1.40093

Supplementary Table 5. Summary of recently reported CO_2RR performances of other reported COF derived electrocatalysts under alkaline conditions (Electrolyte 0.5 M $KHCO_3$).

Electrocatalyst	FE_{CO} (%) at -0.8 V	FE_{CO} (%) at -1.0 V	TOF (s^{-1}) at -1.0 V	j_{CO} (mA cm^{-2}) at -1.0 V	Reference
CoTAPP-PATA-COF	81	67	1.08	15.3	This work
N^+-COF	95	78	1.86	18.1	
NH-COF	94	83	1.74	16.5	
N^+-NH-COF	97	83	2.48	21.4	
CoPc-PI-COF-1	95	~82	4.90	21.2	[2]
COF-300-AR	80	-	-	-	[3]
NiPc-COF	93	~94	1.05	35.0	[4]
COF-367-Co	91	~85	0.50	33.0	[5]
CoP-BDT_{HexO}-COF	98	~90	2.40	10.8	[6]
TAPP(Co)-B18C6-COF	93	71	0.35	9.5	[7]
Co-TTCOF	~88	-	~1.10	~2.5	[8]
TT-Por(Co)-COF	~87	-	~0.10	~5.6	[9]

Supplementary References

[1] T. Lu, F. Chen, *J. Comput. Chem.* **2012**, *33*, 580-592.

[2] B. Han, Y. Jin, B. Chen, W. Zhou, B. Yu, C. Wei, H. Wang, K. Wang, Y. Chen, B. Chen, J. Jiang, *Angew. Chem. Int. Ed.* **2022**, *61*, e202114244.

[3] H. Liu, J. Chu, Z. Yin, X. Cai, L. Zhuang, H. Deng, *Chem* **2018**, *4*, 1696-1709.

[4] M.-D. Zhang, D.-H. Si, J.-D. Yi, S.-S. Zhao, Y.-B. Huang, R. Cao, *Small* **2020**, *16*, 2005254.

[5] S. Lin, S. Diercks Christian, Y.-B. Zhang, N. Kornienko, M. Nichols Eva, Y. Zhao, R. Paris Aubrey, D. Kim, P. Yang, M. Yaghi Omar, J. Chang Christopher, *Science* **2015**, *349*, 1208-1213.

[6] T. He, C. Yang, Y. Chen, N. Huang, S. Duan, Z. Zhang, W. Hu, D. Jiang, *Adv. Mater.* **2022**, *34*, 2205186.

[7] S. An, C. Lu, Q. Xu, C. Lian, C. Peng, J. Hu, X. Zhuang, H. Liu, *ACS Energy Lett.* **2021**, *6*, 3496-3502.

[8] H.-J. Zhu, M. Lu, Y.-R. Wang, S.-J. Yao, M. Zhang, Y.-H. Kan, J. Liu, Y. Chen, S.-L. Li, Y.-Q. Lan, *Nat. Commun.* **2020**, *11*, 497.

[9] Q. Wu, M.-J. Mao, Q.-J. Wu, J. Liang, Y.-B. Huang, R. Cao, *Small* **2021**, *17*, 2004933.
